# Waveguide-integrated metasurfaces for multi-channel crosstalk-free holography

**DOI:** 10.1038/s41377-024-01552-9

**Published:** 2024-08-24

**Authors:** Hyeonsu Heo, Junsuk Rho

**Affiliations:** 1 Department of Mechanical Engineering, Pohang University of Science and Technology (POSTECH), Pohang, Republic of Korea; 2 Department of Chemical Engineering, Pohang University of Science and Technology (POSTECH), Pohang, Republic of Korea; 3 Department of Electrical Engineering, Pohang University of Science and Technology (POSTECH), Pohang, Republic of Korea; 4 POSCO-POSTECH-RIST Convergence Research Center for Flat Optics and Metaphotonics, Pohang, Republic of Korea

**Keywords:** Metamaterials, Nanophotonics and plasmonics, Displays, Optical data storage

## Abstract

Limited information capacity and inter-channel crosstalk in metaholograms hinder their practical use in display applications. Leveraging waveguide-based metasurfaces, the integration of spin and angle-of-incidence multiplexing facilitates the generation of broadband six-channel metaholograms free from crosstalk.

Metaholograms generate holographic images using subwavelength scale structures and have garnered significant interest due to their exceptional characteristics, including compactness, small pixel size, and large viewing angles^1^. They are anticipated to be utilized in futuristic applications such as 3D displays^2,3^, photonic encryption^4^, and AR/VR systems^5,6^. However, metaholograms have struggled with limited information capacity, which hinders their usability in practical applications. Therefore, achieving high information capacity with minimized inter-channel crosstalk is a crucial goal in metahologram design.

To address this issue, researchers have explored various strategies to increase the multiplexing channels of metaholograms, typically by designing metasurfaces to have different responses depending on the different states of the incident light, such as polarization^7^, wavelength^8,9^, angle of incidence^10,11^, and orbital angular momentum (OAM)^12^. Polarization multiplexing has emerged as a dominant strategy, enabling the generation of different holographic images across multiple channels by adjusting the incident polarization. Numerous studies have focused on encoding multiple images using two orthogonal polarization channels, while also demonstrating utility in dynamic holographic displays and sensors through the use of liquid crystals^13,14^. However, the maximum number of independent polarization channels is restricted to three for 2D planar single-layer metasurfaces, even when using off-diagonal elements of the Jones matrix, due to the limited degrees of freedom within the matrix^15^. To further increase the multiplexing channels, engineered noise has been introduced to reduce the correlation between holographic channels, however, it is difficult to generate crosstalk-free images^16^. Another design method involves angle-dependent multiplexing, which encodes different images based on the angular responses controlled by the incident angle of the light^10,11^. Despite these efforts, the limited channel number of metaholograms still remains an unresolved issue, requiring further research on novel design methods to advance the practical use of metaholograms.

Recently, the development of multi-channel holograms using on-chip metasurfaces integrated with optical waveguides has gained interest due to their compatibility with chip-based miniaturized optical systems^5^. Now, writing in eLight, Liu et al. propose waveguide-based multi-channel metaholograms that simultaneously multiplex spin and angle of incidence using a *k*-space translation, as shown in Fig. 1^17^. The central period region of *k*-space, determined by the illumination wavelength (λ_0_) and pitch of the meta-atom (P), can be partitioned into the propagation-wave region and the evanescent-wave region. When the desired image is selectively translated to the propagation-wave region by adjusting the angle of the guided light, it becomes observable in the far-field. The translation distance can be determined by the total internal reflection (TIR) angle (α), and the direction of translation can be controlled by the azimuthal angle (β) of the guided light. As a demonstration, the authors position six different images at the vertices of a regular hexagon in *k*-space (the green dashed line in Fig. 1b), such that each can be moved to the center of the *k*-space through translation induced by corresponding angles of guided light. Additionally, a geometric phase is employed to switch the positions of images between opposite vertices depending on the spin state. Unwanted images remain in the evanescent-wave region and are not projected to the far field, ensuring that only the desired image is observed under the specific incident polarization and azimuthal angle. Consequently, a six-channel crosstalk-free metahologram is achieved.

**Fig. 1:**
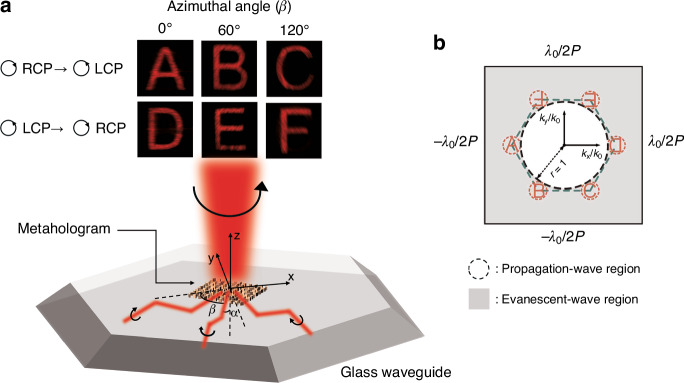
Waveguide-based multi-channel metahologram. **a** Multiple images can be projected without crosstalk depending on the spin state and azimuthal angle (β) of the guided light. **b** In the central period region of *k*-space, only desired images are selectively moved to the propagation-wave region while the undesired crosstalk remains in the evanescent-wave region. Adapted with permission from ref.^17^, copyright 2024, SpringerOpen

Furthermore, the versatility of this design method is demonstrated through other various functionalities. By positioning identical images rotated 180° relative to each other at the opposite vertices of the regular hexagon, a polarization-independent three-channel metahologram multiplexed by the angle of incidence can be generated. Additionally, by leveraging the broadband and dispersive nature of geometric phase metasurfaces, three independent images can be encoded at different wavelengths (red, green, and blue) of illuminated light at different azimuthal angles. Combining this with two-channel polarization multiplexing enables the generation of two independent colored images depending on the incident polarization. Moreover, the authors suggest the potential for further increasing the number of multiplexing channels. By reducing the pitch of the meta-atom and expanding the central period region in *k*-space, more area is secured for encoding a larger number of images, achieving an eight-channel crosstalk-free metahologram. Finally, to realize an even higher capacity metahologram, the authors simulate their design method in combination with OAM multiplexing, increasing the number of channels to eighteen, and demonstrating the potential for further improvements.

In summary, Liu et al. demonstrate broadband multi-channel crosstalk-free metaholograms by effectively combining polarization and angle multiplexing within a waveguide-integrated meta-holography system. Notably, the TIR illumination scheme offers the advantage of preventing disturbance from zero-order diffraction compared to free-space illumination, though it may degrade the working efficiency of the overall holography system. Nevertheless, further optimization of the metasurface using alternative low-loss materials or algorithms such as particle swarm optimization for high-performance meta-atoms could enhance the system efficiency, making high-capacity metaholograms more viable for potential applications. With the growing attention towards developing compact and high-fidelity displays, metaholograms have emerged as promising candidates. This work suggests new possibilities for utilizing on-chip high-capacity metahologram technologies in various future applications, paving the way for the development of advanced compact optical systems.
